# Selection of Reference Genes for Normalization of Gene Expression in *Thermobia domestica* (Insecta: Zygentoma: Lepismatidae)

**DOI:** 10.3390/genes12010021

**Published:** 2020-12-25

**Authors:** Yu Bai, Ya-Nan Lv, Mei Zeng, Pei-Yao Jia, Hu-Na Lu, Yi-Bo Zhu, Sheng Li, Ying-Ying Cui, Yun-Xia Luan

**Affiliations:** Guangdong Provincial Key Laboratory of Insect Development Biology and Applied Technology, Institute of Insect Science and Technology, School of Life Sciences, South China Normal University, Guangzhou 510631, China; yubai@m.scnu.edu.cn (Y.B.); 2019022482@m.scnu.edu.cn (Y.-N.L.); zengmei@m.scnu.edu.cn (M.Z.); 2019022472@m.scnu.edu.cn (P.-Y.J.); 2020022807@m.scnu.edu.cn (H.-N.L.); 2020022824@m.scnu.edu.cn (Y.-B.Z.); lisheng@scnu.edu.cn (S.L.); cuiyingying@m.scnu.edu.cn (Y.-Y.C.)

**Keywords:** *Thermobia domestica*, reference genes, expression stability, quantitative real-time PCR, RNA *interference*

## Abstract

Zygentoma occupies a key evolutionary position for understanding the evolution of insect metamorphosis but has received little attention in terms of genetic analysis. To develop functional genomic studies in this insect, we evaluated five candidate internal reference genes for quantitative RT-PCR (qPCR) studies from *Thermobia domestica*, a representative species of Zygentoma, including *Actin 5C* (*Actin5C*), *Elongation factor-1 alpha* (*EF1A*), *Ribosome protein S26* (*RPS26*), *Ribosome protein L32* (*RPL32*), and *Superoxide dismutase 2* (*SOD2*), at different developmental stages, in various body parts, and under dsRNA microinjection and starvation stresses, using four algorithms (delta Ct, geNorm, NormFinder and BestKeeper) and a comparative algorithm (RefFinder). Specific suitable reference genes were recommended across specific experimental conditions, and the combination of *RPS26* and *RPL32* was appropriate for all tested samples. Employing our selected reference gene combination, we investigated the gene expression pattern of *Myoglianin* (*Myo*), a crucial gene-regulating insect metamorphosis, in ametabolous *T. domestica*, and demonstrated the efficiency of RNA interference (RNAi) in firebrat nymphs. This study provides a basis for reliable quantitative studies of genes and greatly benefits evolutionary and functional genomics studies in Zygentoma.

## 1. Introduction

The basal insect order Zygentoma includes the so-called *silverfish* and *firebrats*. In contrast to winged insects, primitively wingless members of Zygentoma molt throughout their entire lives and do not undergo distinct morphological changes except for increases in size. As the extant ametabolous group closest to pterygote insects [1], Zygentoma occupies a key phylogenetic position for studying the evolution of insect metamorphosis. However, to date, Zygentoma has received little attention regarding genetic analysis. As a potential model organism of ametabolous insects, the firebrat *Thermobia domestica* is widely distributed worldwide. Firebrats are easy to maintain, breed, and synchronize in a laboratory. Rough genomic data from *T. domestica* have been reported recently [2], which facilitates genetic studies of *T. domestica*.

Accurate quantification of gene expression and assessment of gene function by silencing gene expression are needed for successful functional genomics. Quantitative RT-PCR (qPCR) has emerged as a powerful tool to measure gene expression. Many factors influence the accuracy and interpretation of qPCR, including the quantity and quality of the starting material, RNA extraction, cDNA synthesis, and other laboratory procedures. To limit variability, normalization of the data occurs by comparing target gene expression levels to those of reference genes. Additionally, reference genes are assumed to have stable expression across various biotic and abiotic stresses and treatments (e.g., tissues and developmental stages). Depending on the experiment, these assumptions may not be valid; indeed, recent research shows that a condition-specific reference gene for a given species needs to be identified for accurate measurements of gene expression. Because the reliability of qPCR depends largely on the reference gene(s) used to normalize the data, it is crucial to know the stability of the reference gene(s) under specific experimental conditions prior to qPCR. Generally, five statistical algorithms, the delta Ct method, geNorm, NormFinder, Best-Keeper, and RefFinder, are employed to determine the expression stabilities of candidate reference genes. Although the value for *rp49* has been used as a reference to investigate *HR3*, *E75,* and *timeless* (*tim*) gene expression in *T. domestica* with qPCR [3,4], there is no stable reference gene quantification system for *T. domestica*.

RNA interference (RNAi) results in a sequence-specific knockdown of gene expression at the posttranscriptional level, as introduced double-stranded RNAs (dsRNAs) cause the degradation of identical mRNAs. dsRNA can be introduced in different ways, but injection is the most common method to deliver dsRNA into organisms. Ohde et al. [5] attempted microinjection of *Distal-less* (*Dll*) dsRNA into embryos of *T. domestica*, which induced defects in the formation of outgrowths. The Tomioka laboratory has performed molecular analyses of the circadian clock in firebrats by injecting dsRNA of several clock genes into the abdomen of adult firebrats, which effectively abolishes rhythmic expression [3,4]. The postembryonic development period is essential for insect growth and the accumulation of reserves. To date, few RNAi studies on nymph stages in Zygentoma have been conducted.

Here, our goals were to comprehensively evaluate the optimal reference genes for precise quantification of mRNA transcripts of *T. domestica* in different developmental stages and various tissues, as well as under RNAi and starvation stresses, in nymphs of *T. domestica*. Normalized with the selected combination of internal reference genes, we evaluated the spatiotemporal expression and RNAi efficiency of *Myo*, a ligand in the TGF-β signaling pathway [6,7]. These data lay a solid foundation for further genetic studies on *T. domestica* and provide directions for studying the evolution of insect metamorphosis.

## 2. Materials and Methods

### 2.1. Insect Rearing and Sampling Strategy

Firebrats were reared in plastic containers inside a climate chamber (37 °C, ~75% relative humidity, and without light). They were fed commercial rat food, and cotton balls were necessary for them to lay eggs. The insects go through egg, nymph, and adult stages. Most eggs hatch in 12–13 days, and then nymphs undergo two pro-nymph stages (N1–2) lasting 3 days, six nymph stages (N3–8), each requiring 5 days, and a preadult stage (N9) for 7–9 days. After nine nymph instars, the insects molt into sexually mature adults (A1) and pass through 45 to 60 instars undergoing molting and reproductive cycles.

In view of the fact that we usually utilize firebrats after N6 for studies, we chose mixed specimens at nymph stages N6, N7, N8, and N9 and adult stages A1, A3, and A4 to evaluate potential reference genes for different developmental stages. In addition to the whole body (WB), four different body parts, including the head (H), prothorax (T1), mesothorax/metathorax (T23), and abdomen (A), were dissected from each instar. For dissections and tissue sampling, specimens were anesthetized with carbon dioxide. Specimens were immersed in 1ⅹPBS (pH 7.2), and tissues were collected under a stereomicroscope. Approximately 20 Hs, 20 T1s, 10 T23s, 5 As, and 5 WBs at the N6 and N7 stages and 15 Hs, 15 T1s, 8 T23s, 4 As, and 4 WBs at the N8, N9, and A1 stages were collected for the qPCR assay. Similarly, approximately 10 Hs, 10 T1s, 5 T23s, 3 As, and 3 WBs at the A3 and A4 stages were collected for the qPCR assay, and each sample included three biological replicates. All samples were stored inside 1.5 mL RNase-free microfuge tubes at −80 °C until use.

### 2.2. Identification of Candidate Reference Gene Homologs in T. domestica

Based on previous studies of reference genes from other insects [8], five candidate genes, *Actin 5C* (*Actin5C*), *Elongation factor-1 α* (*EF1A*), *Ribosome protein S26* (*RPS26*), *Ribosome protein L32* (*RPL32*), and *Superoxide dismutase 2* (*SOD2*), were selected for the identification of stable reference genes in *T. domestica*. A BLAST search against the transcriptome (our unpublished data) and genome data [2] of *T. domestica* was performed to find homologs of the five genes. Primers specific to each gene were designed individually (https://www.ncbi.nlm.nih.gov/tools/primer-blast/index.cgi?LINK_LOC=BlastHome). The amplification efficiency (*E*) and correlation coefficient values (R^2^) of the primers were calculated by a 4-point standard curve obtained by serial dilutions of known concentrations of cDNA template (Table S1 and Figure S1). The size and sequence of each PCR product were further checked through 1% agarose gel electrophoresis and DNA sequencing.

### 2.3. Total RNA Extraction, cDNA Synthesis, and qPCR

Total RNA was isolated from the prepared samples using RNAiso plus reagents (Takara, Dalian, China) following the supplier’s instructions. The quality and quantity of isolated RNA samples were checked by employing a NanoDrop2000 (Thermo Scientific, Waltham, MA, USA). RNA samples with a 260/280 ratio between 1.80 and 2.0 and a 260/230 ratio between 2.00 and 2.20 were used for qPCR analysis. An aliquot of 800 ng of total RNA was used to synthesize first-strand cDNA using the PrimeScript™ RT reagent Kit with gDNA Eraser (Perfect Real Time) (Takara, Dalian, China). The qPCR experiment was performed in accordance with the Minimum Information Required for Publication of Quantitative Real-time PCR Experiments (MIQE) Guidelines [9]. qPCR was performed on the Applied Biosystems™ QuantStudio™ 6 Flex Real-Time PCR System with three biological replicates and three technical replicates. Each PCR contained 8 µL of 1:20 diluted cDNA, 10 µL of SYBR^®^ Premix Ex Taq™ II (Tli RNaseH Plus) (Takara, Dalian, China), and 1 µL each of forward and reverse gene-specific primers (10 µM). A two-step program (94 °C for 3 min; 40 cycles of 94 °C for 10 s and 56 °C for 30 s) was used for qPCR.

### 2.4. Cloning, Synthesis, and Delivery of dsRNA

Gene-specific paired primers (Table S2) were designed to amplify a 232 bp fragment corresponding to *T. domestica Myoglianin* (*Tdmyo*; obtained from our unpublished transcriptome and verified with the genome data [2]) and a 193 bp fragment corresponding to *Mus* musculus lymphotoxin A (*Muslta*; GenBank: XM_006536550.2) as an unrelated gene absent in *T. domestica*. PCR amplification was performed using TaKaRa Ex Taq. The amplified fragments were retrieved from a 1.2% agarose gel using an AxyPrep DNA Gel Extraction Kit (Axygen Scientific, Union City, CA, USA) and were then cloned into the pGEM-T Easy Vector (Promega, Madison, WI, USA) following the manufacturer’s protocol. All positive clones were finally validated by DNA sequencing.

Prepared plasmids served as templates for dsRNA synthesis. In vitro transcriptional synthesis and purification of dsRNA were performed using a T7 RiboMAX™ Express RNAi System (Promega, Madison, WI, USA). The final concentration was adjusted to 6 µg/µL with a NanoDrop2000, and all dsRNAs were stored at −80 °C until use. RNAi treatments were performed by body microinjection of dsRNA into N8 nymphs. Firebrats were anesthetized with carbon dioxide, and 200 nL of purified dsRNA was injected into the interlaminar fold of the abdomen per insect at a slow speed using a Nanoinject II microinjector apparatus (Drummond Scientific, Broomall, PA, USA). Glass capillaries (3.5 inc 3-000-203-G/X micropipettes, Drummond Scientific, Broomall, PA, USA) were prepared by a P-97 Micropipette Puller (Sutter Instrument Co., Novato, CA, USA) under the following parameters: heat ¼ 428, pull ¼ 200 and VEL ¼ 10. The specimens were collected 72 h after the treatment and subjected to detection of RNAi efficiency using the whole body.

### 2.5. Starvation Treatment

One-day-old N8 nymphs were starved or normally fed for 72 h. To avoid cannibalism, insects were kept individually. The whole bodies of specimens were collected and subjected to RNA extraction, cDNA synthesis, and qPCR analysis. Following dissection, the tissues were stored at −80 °C until RNA was isolated. Three insects were pooled to constitute one biological replicate, and three biological replicates were analyzed for fed and starved *T. domestica*.

### 2.6. Statistical Analysis

The average C_t_ value was calculated based on three biological replicates. The stability of the five candidate reference genes was evaluated using a web-based comprehensive tool (https://www.heartcure.com.au/reffinder/?type=reference), which contained five universal analysis programs: comparative △C_t_ method [10], NormFinder [11], geNorm [12], BestKeeper [13], and RefFinder [14]. GeNorm calculated the ‘M’ value, with a lower value indicating a more stable expression. The NormFinder algorithm utilizes a linear mixed effect model to estimate both intra- and intergroup variations and to rank the candidate reference genes by stability values (SV). BestKeeper uses Ct values of genes to infer their stability, taking into consideration the standard deviation (SD), *p*-value, and correlation coefficient of each gene. Consequently, a lower ‘CV’ value signifies the suitability of a particular gene to serve as a better internal control. As a comprehensive tool, RefFinder integrates the results from geNorm, NormFinder, BestKeeper, and the comparative Ct method and then ranks candidate reference genes based on the geometric mean values (GM).

To determine the optimal number of reference genes for normalization, the pairwise variations V_n/n+1_ (n is the number of reference genes) were calculated between two sequential normalization factors (NF_n_ and NF_n+1_) by geNorm with a cut-off value of 0.15 [12].

## 3. Results

### 3.1. Expression Profiles of Candidate Reference Genes

**Figure 1:**
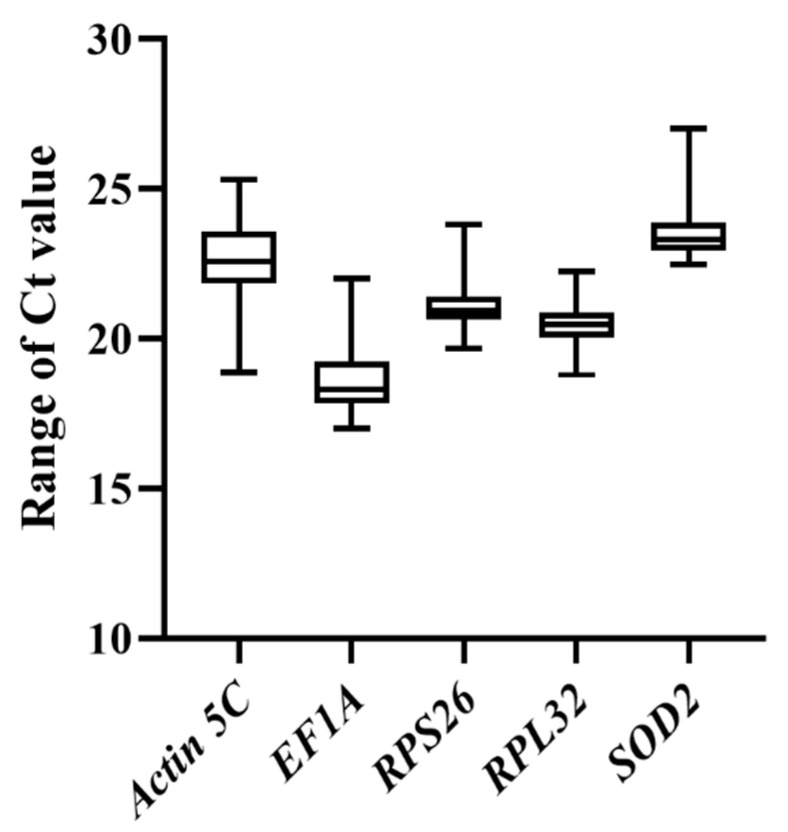
Ct value distribution of five reference genes in all analyzed samples of *Thermobia domestica*. The whiskers of the boxes are the minimum and maximum Ct values, and the horizontal lines inside the boxes are the median values.

Under specific conditions at different developmental stages and in different body parts of *T. domestica*, all calculated Ct values of the five candidate genes varied from 17.00 to 27.01. According to these Ct values, *EF1A* showed the highest expression level, while *SOD2* showed the lowest (Figure 1). Based on the standard deviation (SD) values, *RPL32* showed the lowest expression variations among different samples (mean Ct value ± SD = 20.44 ± 0.64), and *Actin5C* showed the highest expression variations (mean Ct value ± SD = 22.59 ± 1.31) among the five genes. Rankings are shown in Table 1 when the results from all the tested samples were summarized and analyzed by the △C_t_ method, NormFinder, geNorm, BestKeeper, and RefFinder.

### 3.2. Evaluation of Candidate Genes for Gene Expression in Different Body Parts at Specific Developmental Stages of T. domestica

**Figure 2:**
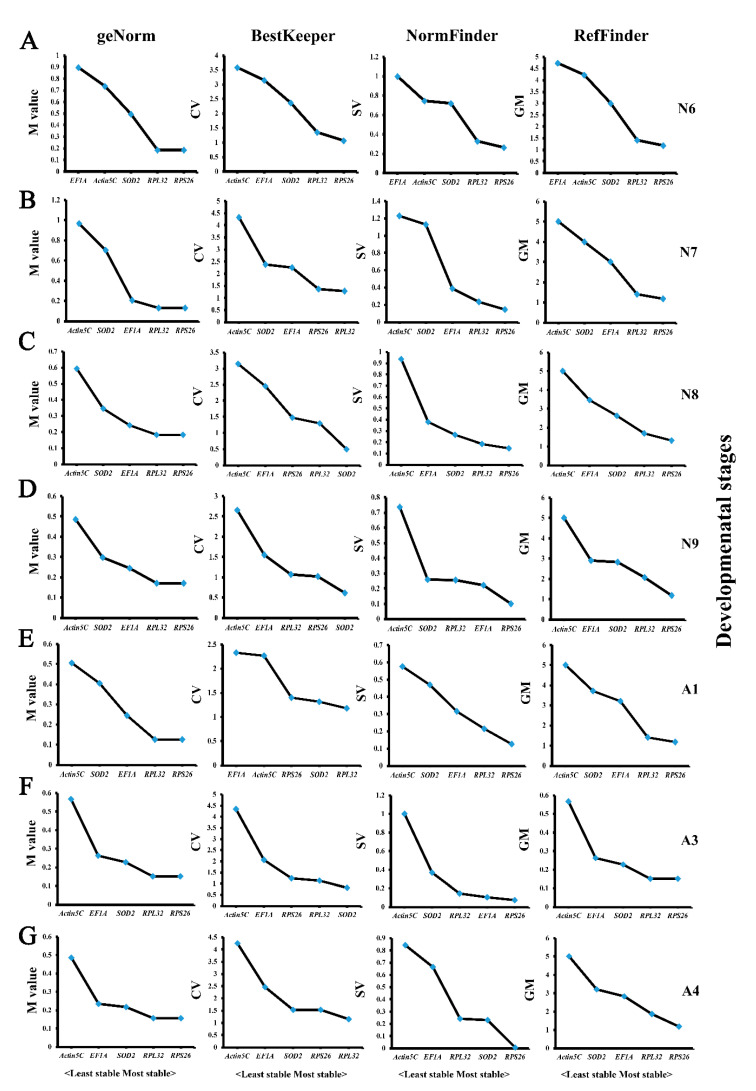
Stable analysis of five candidate genes in different tissues of *Thermobia domestica* (head, prothorax, mesothorax/metathorax, abdomen, and whole body) at the developmental stages of nymphs N6 (**A**), N7(**B**), N8 (**C**), and N9 (**D**), and adults A1 (**E**), A3 (**F**), and A4 (**G**) based on four algorithms.

The expression levels of the five candidate genes in five body parts (H, T1, T23, A, and WB) at selected developmental stages (N6–N9, A1, A3, and A4) were determined by qPCR and analyzed for their expression stabilities by the four statistical algorithms. At developmental stages N6, N7, and A4 of *T. domestica*, geNorm, BestKeeper, and NormFinder analyses obtained consistent results showing that *RPS26* and *RPL32* were the most stably expressed genes in different body parts (Figure 2A,B,G). At developmental stages N8, N9, A1, and A3 of *T. domestica*, the gene expression of *RPS26* and *RPL32* was also most stable based on the geNorm and NormFinder algorithms, but BestKeeper analysis showed that *SOD2* and *RPL32* were the two best choices (Figure 2C–F). Summarized rankings from RefFinder analysis showed that *RPS26* and *RPL32* were the most stably expressed genes in different body parts at all tested developmental stages of *T. domestica*, while the expression of *Actin5C* was the least stable in different body parts at all tested developmental stages except at N6, in which *EF1A* was more altered than *Actin5C* (Figure 2).

### 3.3. Evaluation of Candidate Genes for Gene Expression at Different Developmental Stages in Specific Body Parts of T. domestica

**Figure 3:**
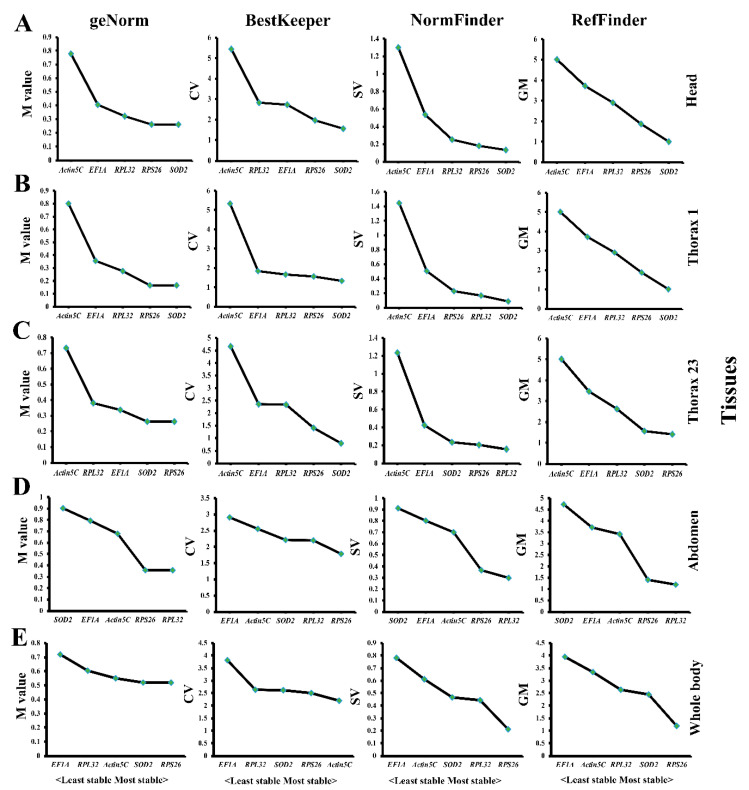
Stable analysis of five candidate genes at different developmental stages from nymphs (N6–N9) and adults (A1, A3, and A4) of *Thermobia domestica* in different tissues: head (**A**), prothorax (**B**), mesothorax/metathorax (**C**), abdomen (**D**), and whole body (**E**) based on four algorithms.

To determine the expression of these candidate genes at different developmental stages in some specific *T. domestica* body parts, we analyzed the expression stability of five candidate genes through qPCR for the developmental stages N6–N9, A1, A3, and A4 in the head, prothorax, mesothorax/metathorax, abdomen, and whole body by using the four algorithms mentioned above. In the head, *SOD2* and *RPS26* were most stable at different developmental stages based on all four analyses (Figure 3A). In the prothorax, *SOD2* and *RPS26* were also optimal according to the geNorm and BestKeeper algorithms, while *SOD2* and *RPL32* were preferred by NormFinder analysis (Figure 3B). In the mesothorax/metathorax and whole body, *SOD2* and *RPS26* were recommended by geNorm and BestKeeper analyses, while *RPL32* and *RPS26* were favored by the NormFinder algorithm (Figure 3C,E); in the abdomen of *T. domestica*, *RPL32* and *RPS26* were selected by all algorithms (Figure 3D). According to the comprehensive ranking analysis of RefFinder, *SOD2* and *RPS26* were the two most stably expressed genes at different developmental stages in the head, prothorax, mesothorax/metathorax, and whole body of *T. domestica*, respectively, while *RPL32* and *RPS26* were the most appropriate reference genes for gene expression studies in the abdomen of *T. domestica*. In addition, the least stable genes at different developmental stages were *Actin5C* in the head, prothorax and mesothorax/metathorax, *SOD2* in the abdomen, and *EF1A* in the whole body.

### 3.4. Evaluation of Candidate Genes for Gene Expression in Tdmyo-Silenced and Starvation Conditions

**Figure 4:**
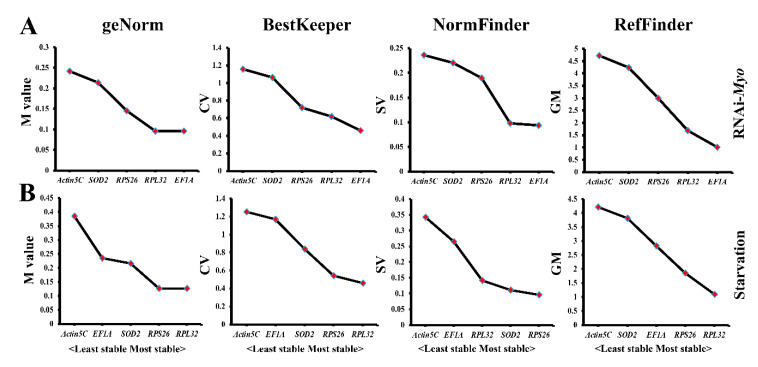
Stable analysis of five candidate genes under RNAi-*Myo* (**A**) or starvation stresses (**B**) based on four algorithms.

To determine whether RNAi affects the expression of the five candidate genes, we silenced *Myo* expression in *T. domestica* through microinjection and verified the *Myo* expression level by qPCR analysis. *Muslta*, a gene absent in *T. domestica*, was used as the control. Analysis of the gene expression of five candidate genes based on the qPCR data from the whole body samples injected with dsRNA of *Tdmyo* or *Muslta* showed that *EF1A* and *RPL32* were the most stably expressed genes using the geNorm, BestKeeper, NormFinder, and RefFinder algorithms (Figure 4A).

To further evaluate the effect of starvation stress, we compared the expression of five candidate genes in *T. domestica* whole body samples under fed or starved conditions through qPCR. Analyses with the geNorm and BestKeeper algorithms showed that *RPL32* and *RPS26* were the most stably expressed genes under starvation stress, but *RPS26* and *SOD2* were optimal using NormFinder analysis. The comprehensive stability ranking of the five candidate genes generated by the RefFinder algorithm was *RPL32* > *RPS26* > *EF1A* > *SOD2* > *Actin5C* (Figure 4B).

### 3.5. Determination of the Optimal Number of Reference Genes Needed for qPCR Normalization

To reduce the probability of biased normalization, multiple reference genes have been used to normalize target gene expression [15,16,17]. However, either too few or too many reference genes may be detrimental to the accuracy of target gene expression [18,19]. Based on the evaluated rankings of all experimental sets in Figure 2 and Figure 3, the pairwise variation values between ranked genes (V_n/n+1_) were calculated by the geNorm algorithm to demonstrate the effect of adding reference genes on the stability of the normalization factor, and to determine the optimal number of reference genes in *T. domestica* using a cutoff of 0.15 [12]. A threshold value below 0.15 indicated that the additional reference gene had no significant improvement on normalization in qPCR data.

**Figure 5:**
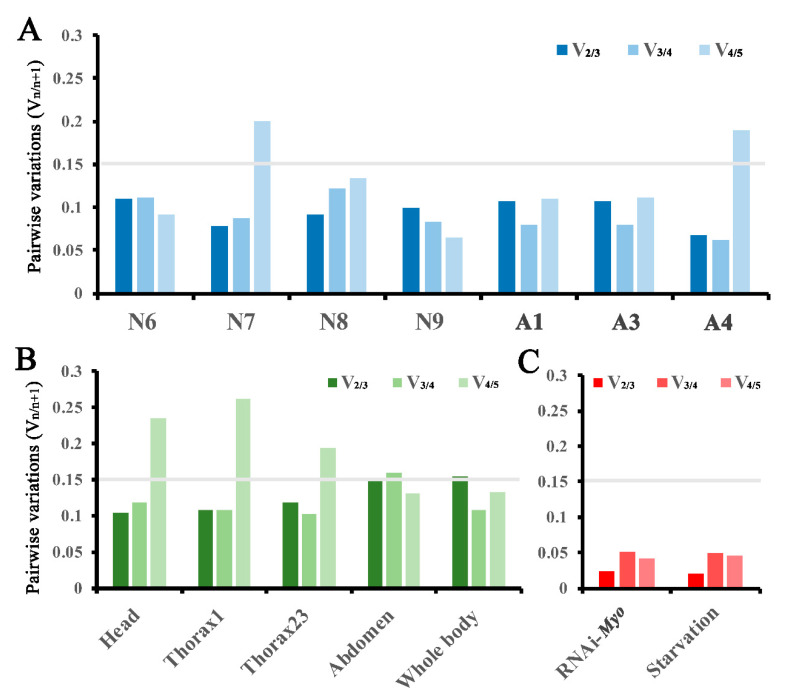
Evaluation of the optimal number of reference genes for normalization in *Thermobia domestica.* The pairwise variations (V _n/n+1_, n is the number of reference genes) was calculated between two sequential normalization factors (NF_n_ and NF_n+1_) by geNorm with a cut-off value of 0.15 [12] using data: (**A**) from different tissues at the developmental stages of N6–N9, A1, A3, and A4; (**B**) at different developmental stages in the head, prothorax, mesothorax/metathorax, and abdomen; (**C**) in whole insects at the 8th nymphal instar under RNAi and starvation treatments.

At each studied developmental stage of nymphs (N6–N9) and adults (A1, A3, and A4), the V_2/3_ value for all experimental sets in different tissues was lower than 0.15 (Figure 5A), suggesting that two reference genes can standardize these samples well. According to the rankings in Figure 2, *RPS26* and *RPL32* together were the optimum reference gene combination for qPCR analysis in different body parts at the selected stages of middle and late nymphs (N6–N9) and adults (A1, A3, and A4). In contrast, *Actin5C* introduced negative effects at stages N7 and A4, since their V_4/5_ values increased obviously.

In the five investigated body parts, the V_2/3_ values at different developmental stages were apparently lower than 0.15 in the head, prothorax, and mesothorax/metathorax of *T. domestica* and were very close to 0.15 in the abdomen and whole body (Figure 5B). Thus, two reference genes would be effective for standardizing these samples. Based on the evaluation ranking in Figure 3, the combination of *SOD2* and *RPS26* was the optimum for qPCR analysis at different stages in the head and thorax, the combination of *RPL32* and *RPS26* was ideal in the abdomen, and the combination of *RPS26* and *SOD2* was a suitable normalization in the whole body. Additionally, the inclusion of *Actin5C*, the least stable gene in the head, prothorax, and mesothorax/metathorax, greatly increased the corresponding V_4/5_ values in each tissue.

Under the *Tdmyo*-silenced and starvation conditions, all tested combinations were lower than 0.05, and both V_2/3_ values were the lowest (Figure 5C). Based on the evaluation ranking in Figure 4, the combination of *EF1A* and *RPL32* for RNAi-*Tdmyo* and the combination of *RPL32* and *RPS26* for starvation treatment were the best choices.

Overall, the combination of *RPL32* and *RPS26* was the best choice for most tested samples, except for samples at different stages in the head, thorax, and whole body (*RPS26* and *SOD2*), and under RNAi-*Tdmyo* treatment (*EF1A* and *RPL32*). However, the inclusion of *RPL32* at different stages in the head, thorax, and whole body had no significant effect on the corresponding pairwise variation (Figure 5B), neither did the inclusion of *RPS26* under RNAi-*Tdmyo* treatment (Figure 5C). Using the same reference standard for all the studies would be best to be able to compare data. Thus, the combination of *RPS26* and *RPL32* is recommended for all tested experimental conditions.

### 3.6. Normalization of qPCR Data of Tdmyo Using Selected Reference Genes

**Figure 6:**
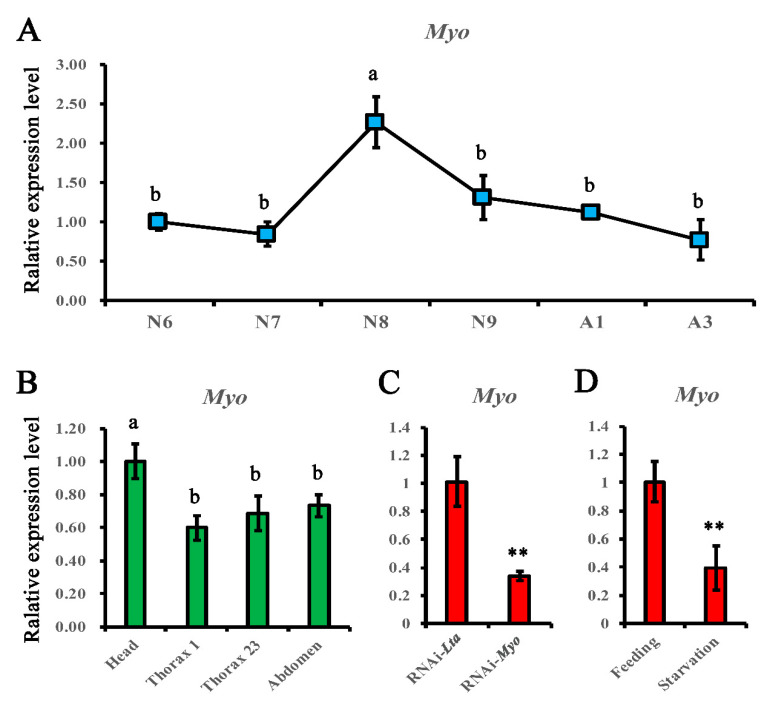
Relative expression levels of *Tdmyo* under various experimental conditions normalized with *RPS26* and *RPL32*. Error bars show the mean standard error calculated from three biological replicates. (**A**) In the head of *T. domestica* from the middle nymphs to adults. (**B**) In four body parts from individuals at the 8th nymphal instar. (**C**) In whole insects at the 8th nymphal instar under RNAi treatment. (**D**) In whole insects at the 8th nymphal instar under feeding or starvation conditions. Different letters (a, b) in (**A**,**B**) indicate significant differences of values (ANOVA, LSD, *p* < 0.05). The statistical level in (**C**,**D**) was assessed with ** *p* < 0.01.

*Myo* has been proven to trigger the *premetamorphosis stage* in hemimetabolan insects [7], and it is worth studying *Myo* expression in ametabolous insects to understand its genetic evolution. Normalized with *RPS26* and *RPL32*, the relative expression level of *Tdmyo* in the head of *T. domestica* was similar at N6 and N7, increased remarkably and reached the highest level in N8 (the penultimate nymphal instar), was slightly lower but remained high in N9 (the last nymphal instar), and then progressively decreased in adults (Figure 6A). At N8 of *T. domestica*, the head showed the highest *Myo* mRNA levels, lower *Myo* expression was indicated in the abdomen and mesothorax/metathorax, and the lowest expression was observed in the prothorax (Figure 6B).

Under RNAi conditions at the 8th instar of *T. domestica*, the *Tdmyo* mRNA level was significantly reduced in *dsTdmyo*-treated *firebrats* compared with *dsMuslta*-treated firebrats (Figure 6C), which suggested a high efficiency of RNAi *Myo* in firebrat nymphs.

Similarly, *Myo* mRNA levels were significantly downregulated in the starvation treatment group compared with the fed groups (Figure 6D), demonstrating that nutrition can notably affect *Myo* expression.

## 4. Discussion

qPCR is an efficient and convenient method for gene expression analysis, and using an appropriate reference gene or genes is the basic requirement to achieve qPCR reliability. To date, the selection of reliable reference genes under different conditions has been performed in many insect and mite species [8] such as locusts [20,21], pea aphids [22], psocids [23], honey bees [24], wasps [25], cotton bollworms [26], flies [27,28], silkworms [29], moths [30], beetles [31,32,33] and spider mites [34,35,36], but not the firebrat *T. domestica*, a representative basal insect, which plays a key role in understanding the origin of insect metamorphosis. To develop functional genomic studies in this insect, it is essential to select optimal reference genes and test the efficacy of RNAi. In this study, we comprehensively evaluated the expression of five candidate genes (*Actin5C*, *EF1A*, *RPS26*, *RPL32,* and *SOD2*) in *T. domestica* under various conditions, including in different tissues at some specific developmental stages, at different developmental stages in several specific tissues, and under dsRNA microinjection and starvation stresses. Most rankings obtained from different algorithms (BestKeeper, geNorm, NormFinder, and RefFinder) were consistent, which demonstrated the reliability of our results.

*Actin*, a traditional *housekeeping gene*, has been regarded as a stable reference gene across insect species in various experimental settings [20,21,24,37] but not in beetles [31,32,33]. *Elongation factor 1 alpha* (*EF-1α*), an evolutionarily conserved GTPase, was evaluated as the stable gene in Orthoptera [21], *Drosophila* [37], and Hymenoptera [38]. However, in this work, both *Actin5C* and *EF-1α* were ranked as the two least stable reference genes under most experimental conditions (Figure 2 and Figure 3). In particular, the pairwise variation values were increased greatly by introducing *Actin5C* (Figure 5) in different tissues at the 7th nymphal stage and the 4th adult stage, as well as at different developmental stages in the head, prothorax, and mesothorax/metathorax (Figure 4). Our analyses proposed that *Actin5C* and *EF-1α* were not appropriate to reference genes in *T. domestica*.

Various ribosomal protein genes have been evaluated and showed highly stable expression in insects [8] and mites, such as *RPS9* and *RPL13* in the spider mite [35,36], *RPS18* in honey bee [24], the spider mite [34], bloodsucking *bug* [39], *RPL18* in the bed bug [40], and *RP49/RPL32* in the desert locust [20]. Our results suggested that the *RPS26* and *RPL32* genes were the two most stable genes among the five candidates in different tissues at all developmental stages tested (nymphs and adults) in *T. domestica* (Figure 2). In addition, both *RPS26* and *RPL32* were more stable than *Actin5C* and *EF-1α* at different developmental stages in all studied tissues. *SOD2* performed well at different developmental stages in the head, prothorax, mesothorax/metathorax, and whole body of *T. domestica* but worst in the abdomen (Figure 3). Our results further prove the importance of selecting stable reference genes before each qPCR under specific experimental conditions. Additionally, our pairwise variation analyses suggested that two reference genes, *RPS26* and *RPL32*, can standardize all tested samples of *T. domestica* well.

The postembryonic development period is essential for insect growth, accumulation of reserves, and metamorphosis. To date, there are few RNAi studies on nymph stages in Zygentoma. This study demonstrated a high RNAi efficiency of *Myo* in firebrat nymphs. The *Tdmyo* mRNA level was significantly reduced in *dsTdmyo*-treated *nymphs* at the 8th instar (Figure 6C). *Myo* is a key trigger of the transition from the penultimate to final (premetamorphic) nymphal stage in the hemimetabolan cockroach [7]. With normalization to the selected internal control genes, *RPS26* and *RPL32*, we performed qPCR to study *Myo* expression patterns in firebrat heads at different developmental stages and in different tissues at the 8th nymphal instar, as well as *Myo* expression changes under RNAi-*Myo* and starvation treatments. In the head of *T. domestica*, the highest expression was observed in N8 (Figure 6A), the penultimate nymphal instar, in accordance with the expression pattern in the cockroach [7], indicating its conserved function in hemimetabolous cockroach and ametabolous firebrat.

## 5. Conclusions

In summary, we evaluated five candidate reference genes in *T. domestica*, a representative ametabolous insect, under various experimental conditions. Similar rankings of five candidate genes were obtained by different algorithms, and the optimal number of reference genes was calculated as two for most experimental sets. The top two stable reference genes were as follows: (1) *RPS26* and *RPL32* in different body parts at all tested developmental stages, (2) *SOD2* and *RPS26* at different developmental stages in the head, prothorax, mesothorax/metathorax, and whole body, (3) *RPS26* and *RPL32* at different developmental stages in the abdomen, (4) *RPL32* and *EF1A* under microinjection of dsRNA (*Myoglianin*, *Myo*), and (5) *RPS26* and *RPL32* under starvation stress. Overall, the combination of *RPS26* and *RPL32* was recommended as a suitable reference for all settings performed in this study. In contrast, the expression of *Actin5C* was least stable in most tested samples. By exploring qPCR and RNA*i* in *T. domestica*, we found that *Myo* in wingless *T. domestica* had a similar spatial and temporal expression patterns with those of winged insects, and RNAi effect is prominent in the nymphal stage. Our findings not only help to establish a more accurate and reliable normalization of real-time qPCR data in *T. domestica* but also lay a solid foundation for further studies of RNA interference and gene transcription in basal apterous insects.

## Figures and Tables

**Table 1:** Expression stability ranking of the selected candidate reference genes using Delta Ct, geNorm, BestKeeper, NormFinder, and RefFinder.

Rank	Delta Ct			geNorm		BestKeeper		NormFinder		RefFinder	
	Gene Name	Average Ct	SD	Gene Name	M	Gene Name	CV	Gene Name	SV	Gene Name	GM
1	*RPS26*	21.08	0.75	*RPS26*	0.39	*RPL32*	2.48	*RPS26*	0.27	*RPS26*	1.19
2	*RPL32*	20.44	0.83	*RPL32*	0.39	*RPS26*	2.49	*RPL32*	0.46	*RPL32*	1.41
3	*EF1A*	18.61	0.91	*EF1A*	0.62	*SOD2*	3.39	*EF1A*	0.63	*EF1A*	3.22
4	*SOD2*	23.68	0.96	*SOD2*	0.74	*EF1A*	4.48	*SOD2*	0.69	*SOD2*	3.72
5	*Actin5C*	22.58	1.24	*Actin5C*	0.94	*Actin5C*	4.68	*Actin5C*	1.12	*Actin5C*	5.00

Note: SD, standard deviations; M, global gene expression stability values; CV: coefficient of variation; SV, stability values; GM, geometric mean.

## Supplementary Materials

The following are available online at https://www.mdpi.com/2073-4425/12/1/21/s1, Figure S1: Melting curves, melting peaks and amplification curves of the five selected candidate reference genes. Table S1: Primers of the candidate reference genes used in this study. Table S2: Primers used for qRT-PCR analysis of *Myo* gene expression and dsRNA synthesis.
