# Supplementary material for: Selection of Reference Genes for Normalization of Gene Expression in Thermobia domestica (Insecta: Zygentoma: Lepismatidae)
Source: Genes (Basel). 2020 Dec 25;12(1):21. doi: 10.3390/genes12010021 (PMC7823838; doi:10.3390/genes12010021)
Supplement: Supplementary file 1 [file genes-12-00021-s001.pdf]

Figure S1. Melting curves, melting peaks and amplification curves of the five selected candidate reference genes.

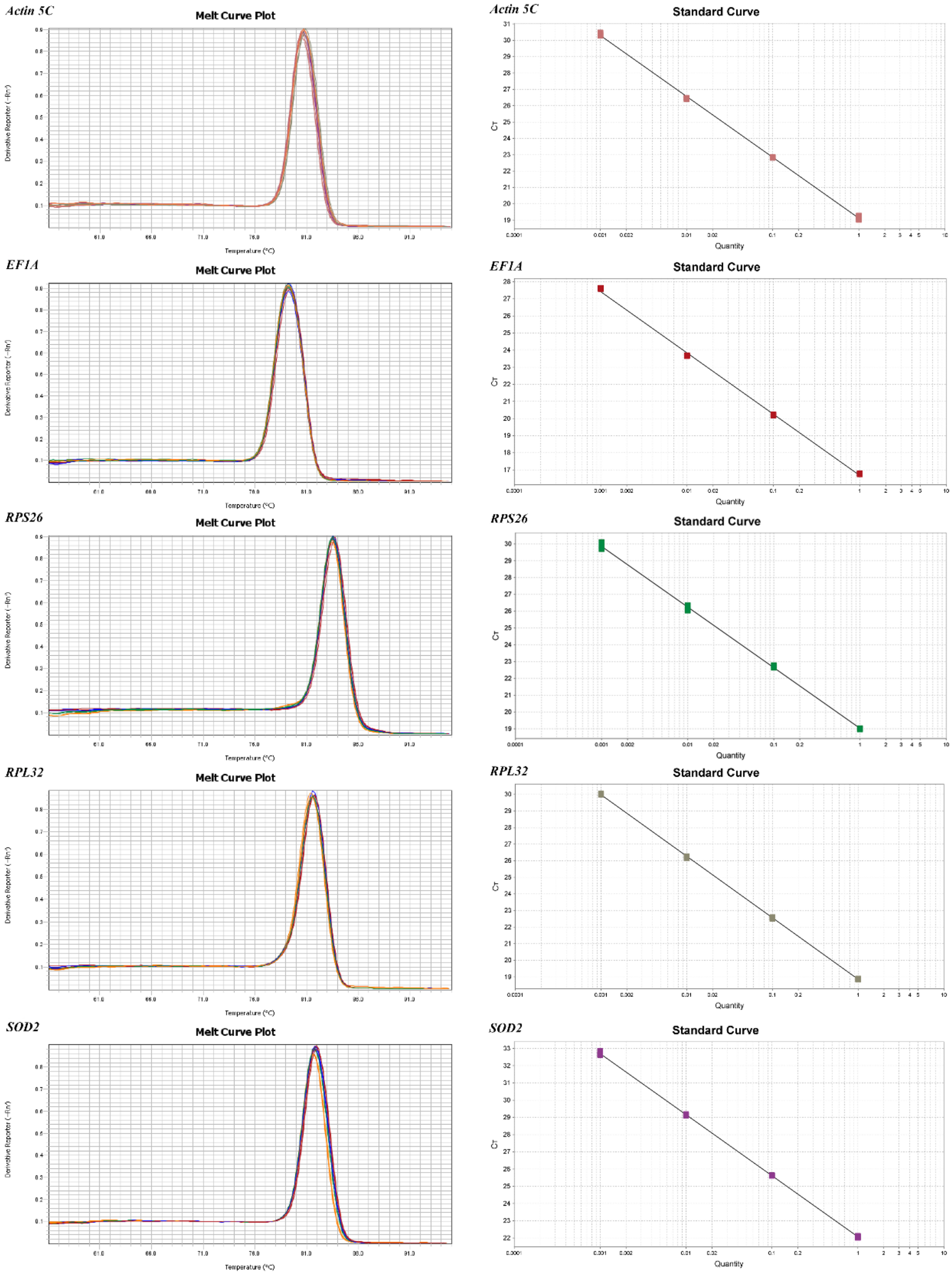

Table S1 Primers of the candidate reference genes used in this study.

Gene Name	Gene Symbol	Primer Sequences (F and R)	Amplicon Length (bp)	Amplification efficiency (%)	R^2
Actin 5C	*Actin5C*	GTATGCTTCTGGACGTACT<br>GCAGAGCATAACCTTCGTAA	90	85.554	0.999
Elongation factor-1 alpha	*EF1A*	ACTGAACCACCTTACAGTGA<br>AACAGCAGCTGGATTGTAAC	93	90.204	0.999
Ribosome protein S26	*RPS26*	ATGACTTGCAAGCGAAGAAA<br>CAGTTTGTACACCTCACTGG	77	89.314	0.999
Ribosome protein L32	*RPL32*	CAGTTTCTGATGCCCTCAAT<br>ACATTGTGAACAAGCACCTT	92	86.062	1
Superoxide dismutase 2	*SOD2*	GGCTTGGCTTGGTTACAATA<br>CAGTTGATGGTTCCAAAGGA	83	91.562	1

Table S2 Primers used for qRT-PCR analysis of *Myo* gene expression and dsRNA synthesis.

Gene Name	Primer Sequences (F and R)	Amplicon Length (bp)	Application
Myo	T7 + GACAGATGGGAGGAAACATCG<br>T7 + TTGTGGGCTTGCTTGTTGAA	232	RT-PCR
Muslta	T7 + CACCCTCTCCACGAATTG<br>T7 + TAGAAGATGCTGCTGTTTCA	193	RT-PCR
Myo	CCCGAGAAAAATGTCGTCTAT<br>CCGATCCACTACCATTCCT	91	qRT-PCR

T7 RNA polymerase promoter sequence: TAATACGACTCACTATAGGG
